# Supplementary material for: Factory-Calibrated Continuous Glucose Monitoring Systems in Type 1 Diabetes: Accuracy during In-Clinic Exercise and Home Use
Source: Sensors (Basel). 2023 Nov 18;23(22):9256. doi: 10.3390/s23229256 (PMC10675113; doi:10.3390/s23229256)
Supplement: Supplementary file 1 [file sensors-23-09256-s001.zip › sensors-2624485-supplementary.pdf]

Supplementary Materials

Table S1: MARD and PEGA results for the in-clinic visit using plasma glucose as reference

Settings	Variable	CGM			P-value
		Dexcom G6	Freestyle Libre 2	Guardian 4	Overall
In-clinic exercise period	**MARD % ± SD**	19.3±9.4	19.3±15.0	14.4±9.7	0.42
	**PEGA AB%**	93.8	91.8	94.6	0.32[CQ]
In-clinic postexercise period	**MARD % ± SD**	16.6±10.2	17.2±11.3	10.9±8.6	0.14
	**PEGA AB %**	96.1	95.2	96.7	

Mean Absolute Relative Difference (MARD), Standard deviation (SD), and Point Error Grid Analysis (PEGA) in zone AB for three continuous glucose monitoring systems (CGM)—Dexcom G6, Freestyle Libre 2, Guardian 4—using plasma glucose measurement as a reference. CQ: Cochran's Q test for heterogeneity in the proportion of PEGA AB between the three sensors.

Figure S1: Point Error Grid for the in-clinic visit using plasma glucose as reference

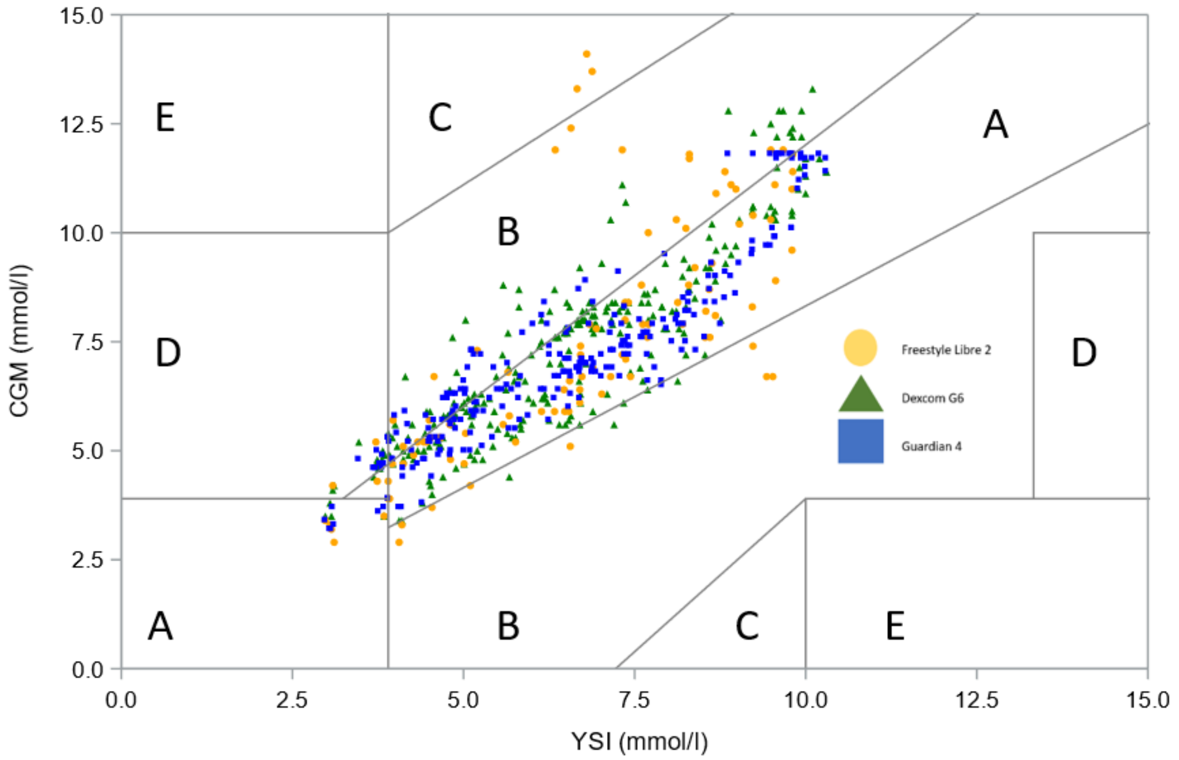

Figure S1: Point Error Grid Analysis of three continuous glucose monitoring systems (CGM: Freestyle Libre 2 = yellow, Dexcom G6 = Green, Guardian 4= Blue) using plasma glucose measurement (YSI) as a reference.

Figure S2: Bland–Altman Plot and Pearson´s regression for the CGM devices

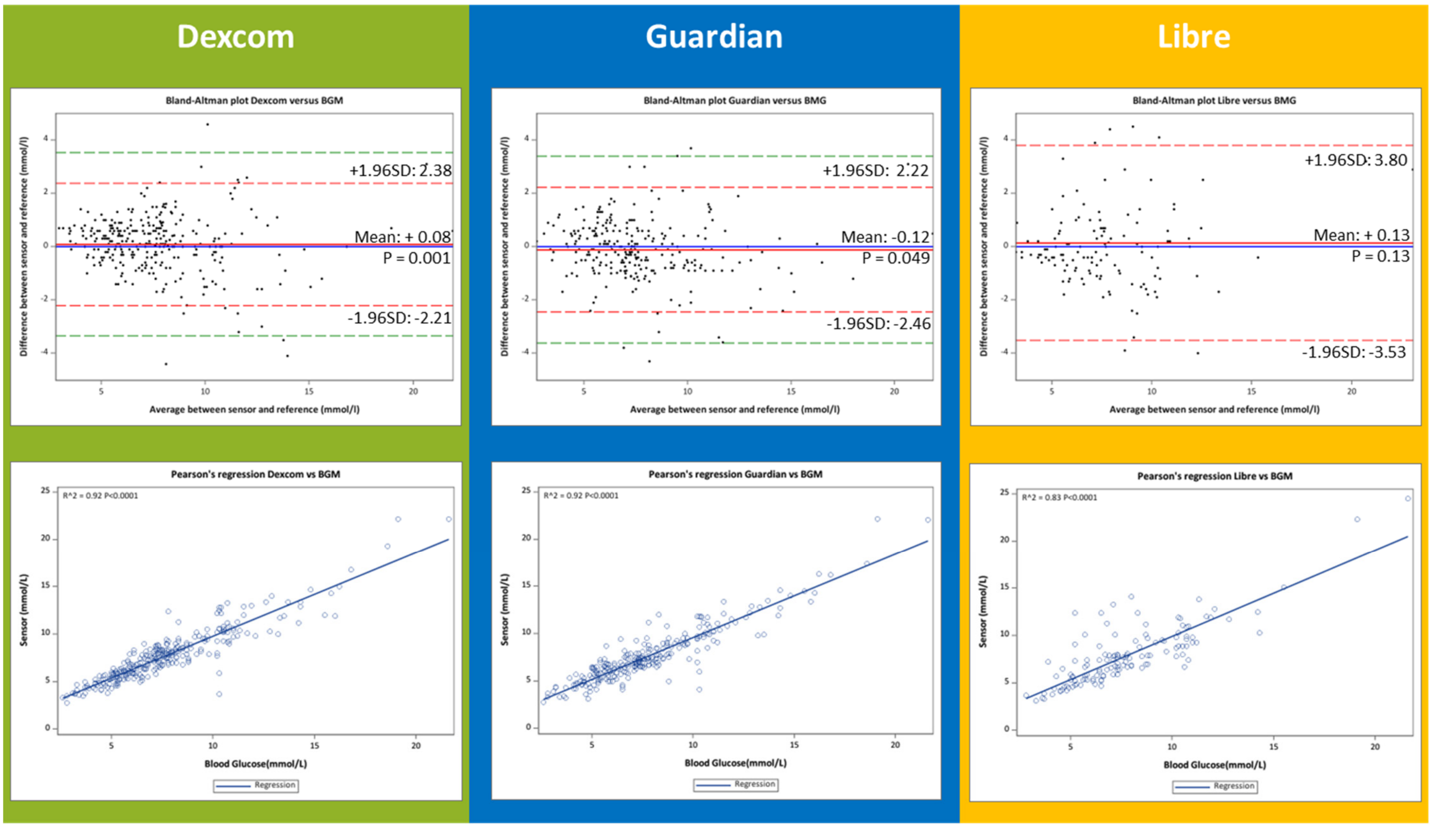

Figure S2: The upper panel are Bland–Altman plots and the lower panel contain scatterplots.
